# Sources of Discomfort in Persons with Dementia: Scale and Initial Results

**DOI:** 10.1155/2015/732832

**Published:** 2015-06-21

**Authors:** Jiska Cohen-Mansfield, Khin Thein, Marcia S. Marx, Maha Dakheel-Ali, Barbara Jensen

**Affiliations:** ^1^Minerva Center for the Interdisciplinary Study of End of Life, Tel-Aviv University, 69978 Tel-Aviv, Israel; ^2^Department of Health Promotion, Sackler Faculty of Medicine and Herczeg Institute on Aging, Tel-Aviv University, 69978 Tel-Aviv, Israel; ^3^Innovative Aging Research, 807 Horton Drive, Silver Spring, MD 20902, USA

## Abstract

The Sources of Discomfort Scale (SODS) assesses discomfort manifestations based on source of discomfort, thus making it both distinct from and complementary to pain assessments for persons with dementia. Sources were categorized as pertaining to physical discomfort, to body position, and to environmental sources. Body position sources of discomfort were related to poor functional status and to pain. The SODS scores were not related to cognitive functioning, and sources of discomfort other than those pertaining to body position were not correlated with pain. This paper demonstrates a direct and enhanced method to detect the manifestations of discomfort separately from pain indicators in a population with advanced dementia. The determination of the source of discomfort has direct implications for intervention.

## 1. Introduction

Pain is defined as “localized physical suffering associated with bodily disorder (as a disease or an injury)” [1]; discomfort is defined as “mental or physical uneasiness” [1]. While the distinction between pain and discomfort is often blurred, the constructs can be distinguished [2, 3]. In a study of patients after orthopedic surgery, descriptions of pain were more often of an internal experience, whereas discomfort was more likely recounted as an environmental stimulus [3]. Pain may also describe a more extreme sensation than discomfort. There is a lack of clear distinction between these constructs in persons with dementia (PWD), as measurements of pain and of discomfort are often used interchangeably.

Three types of discomfort assessments have been reported. The first involves rating scales such as the Discomfort Scale for Alzheimer's disease [4], which is the most commonly used assessment, with indications such as noisy breathing, negative vocalization, sad or frightened facial expressions, relaxed or tense body language, and fidgeting. While this scale has been used to assess discomfort [5, 6], it has also been used to assess pain [7]. Another rating scale is the Discomfort Behavior Scale, with items such as repetitive verbalizations, crying, or tearfulness [8]. Still another assessment is the Disability Distress Assessment Tool (DisDAT, [9]), which requires the caregiver to discern signs of states of being content and in distress. The second type of discomfort assessment involves videotaping PWD with two cameras simultaneously and using specialized software, the Digital Discomfort Labeling Tool (DDLT), to capture the symptoms included in discomfort rating scales [10]. Finally, there is a protocol for assessment of discomfort which includes a physical assessment (e.g., physical causes of discomfort) and an assessment of agitated behaviors, such as physically aggressive behavior or socially inappropriate behaviors [11]. Measures of pain in older PWD are often based on behavioral observations (see reviews, [12, 13], including those obtained by video, [14]). Established pain measures include the Pain Assessment in Noncommunicative Elderly Persons (PAINE, [15]), the Pain Assessment in Advanced Dementia Scale (PAINAD, [16]), and the Pain Assessment Checklist for Seniors with Limited Ability to Communicate (PACSLAC, [17]). Other informant-based pain assessments include the Pain Assessment for the Dementing Elderly (PADE, [18]) and the Noncommunicative Patient's Pain Assessment Instrument (NOPPAIN, [19]).

Discomfort can originate from physical health problems, internal conditions, or environmental conditions [4, 20]. A previous publication [21] noted the high rates of discomfort found in this study. In this paper, we describe the Source of Discomfort Scale (SODS [21]), the sources of discomfort by subtype, and the relationship between sources of discomfort and cognitive, functional, and pain variables. Our focus is based on the following premises:

- Examination of symptoms included in previously published discomfort scales may reveal focus on pain at the expense of the detection of discomfort.
- Some discomfort may not be manifested in discomfort behaviors but may be evident through observation (e.g., a very uncomfortable position) or other assessment (e.g., having cold hands).
- Focusing on the source of discomfort can help caregivers identify the needed intervention. Early detection of discomfort and the ensuing care are essential for assuring quality of life for PWD.

In this paper, we divide the sources of discomfort into three types: physical discomfort, discomfort related to body position, and environmental discomfort. We also split the items on the SODS into those which are observational and those that are or may be based on verbal responses of the participants. We pose the following hypotheses:

1. Verbal items will be correlated with measures of cognitive function (MMSE and items from the MDS), but the observational items will not. We will explore the relationship of the total SODS to measures of cognitive function. This is important as most prior measures of pain and discomfort have been biased in that they were more likely to detect occurrences in persons with higher cognitive functioning.
2. Body position indicators will be correlated with poorer ADL, as persons with higher levels of ADL will be more likely to be able to shift their body position to a more comfortable position.
3. Physical discomfort will be more closely related to pain than other types of discomfort, since those are sensations more internal to the person and less affected by the environment.

## 2. Materials and Methods

### 2.1. Participants

Participants were 179 Nursing Home (NH) residents from six nursing homes in Maryland, USA. Discomfort of the residents was observed as part of the study for the Treatment Routes for Exploring Agitation (TREA) [22] that received IRB approval of the Charles E. Smith Life Communities. Participating residents were selected by the nursing staff of the units of the nursing homes. Participants had been identified by nursing staff as agitated at least several times a day, had a dementia diagnosis, had a Mini Mental State Examination (MMSE) score <25, did not have a bipolar disorder or schizophrenia diagnosis, and were aged 60 and above. Participants' characteristics are described in Table 1. The description of the study was sent to the participant or to the person responsible for consent. With the exception of four residents who provided consent themselves, written consents were provided by a guardian, power of attorney, or family member.

### 2.2. Procedure

The Source of Discomfort Scale (SODS) was completed by several trained research assistants who directly observed each participant over three days (13 hours a day, for three minutes every half hour). The MMSE was administered to each participant, and the Pain Assessment in Noncommunicative Elderly Persons (PAINE) was administered to frontline caregivers by a trained research assistant. These assessments were conducted on different days and often by different research assistants. Background and medical data and data from the Minimum Data Set (MDS [23], Activities of Daily Living, long term memory, and ability to understand others) were obtained from charts kept by NH staff.

### 2.3. Assessments

Background data (age, gender, marital status, ethnicity, and education), medical data pertaining to prescribed medications (including pain relievers and psychotropics), hearing and/or vision impairment, diagnoses (including a dementia diagnosis confirmed by a physician), and Activities of Daily Living (ADL) from the MDS data were obtained from the residents' charts. Cognitive functioning was assessed by the MMSE [24].

Pain was assessed for 89 of the participants who were part of the treatment group for the original study [22] through administering the PAINE [15] to the participants' direct care caregivers from the nursing staff. The PAINE assessment includes 22 items, all of which were based on nursing staff reports of the signs and symptoms they associate with pain in PWD [25]. These include symptoms such as moaning, rigidity, facial grimaces, and restlessness/repetitive movements, as well as clinical indicators such as trembling/shaking, swollen joints, and tight/swollen belly. PAINE was selected because of its superiority over many observational pain scales [26]. The reliability and validity of PAINE have been described elsewhere [15]. The PAINE has been shown to be useful in detecting pain which responds to analgesic intervention in PWD [26].

*The Source of Discomfort Scale (SODS)* was developed on the basis of our prior experiences observing PWD and noticing different types of discomfort. Ability to communicate is progressively diminished in persons with dementia as the dementia advances. This tool is meant for persons who cannot easily and clearly express their sensations, such as discomfort. The research assistant completed items regarding the signs of discomfort. The SODS items were classified into the following categories:

1. * Physical*
  - * Hunger/thirst*: the research assistant offered food and watched the reaction. Is he/she eating all of it without prompting? Similarly, the research assistant offered some water and noticed the response. They also noted if the person requested food or drink.
  - * Rash/fungus*: seeming to try to scratch a body part, excessive touching of clothing.
  - *Constipation*: chart examination for constipation was carried out.
  - *Sleepiness or tiredness*: seeming to be excessively sleepy or tired (note: this was determined using the reasonable person test, which is the idea that any usual person would feel uncomfortable in that position or circumstances). The research assistant rated this as a source of discomfort if the person was not in a comfortable body resting position while seen as sleepy or tired (e.g., this was not rated if the person was sleeping in their own bed).
  - *Feeling uncomfortable*: the research assistant asked the person if he/she was feeling comfortable, and a negative response was noted.
  - *Bathroom*: resident asking to go to the bathroom.
2. * Body positioning*
  - * Seating*: moving in the seat, head lying unsupported, leg dangling, leg stuck in the wheelchair or another piece of furniture, other body parts looking uncomfortable using the reasonable person test, and sitting in the same place without movement for over two hours (note: although each resident was observed directly for short periods of time, research staff stayed on the unit observing other residents and were therefore able to notice movements).
3. * Environmental sources*
  - * Lighting*: insufficient lighting is provided.
  - * Being cold/hot*: complaints of being hot or cold; the research assistant noted whether the participant's hand was excessively cold or hot upon handshaking; the research assistant also used his/her own judgment as to whether the room temperature was excessively cold or hot.
  - * Furniture*: furniture standing in the way of the resident.
  - * Restraints*: resident restrained.
  - * Other residents*: another resident is bothering the resident.

The Pearson correlation between the total number of sources of discomfort across different observation shifts as rated by the two raters who had completed the most SODS assessments was *p* = .51, *p* < .01. The exact frequencies of each item can be found elsewhere [21].

In addition, 6 items were characterized as always or sometimes relying on verbal responses by the resident. These are asking the person if he/she is comfortable, hunger, thirst, complaints of being hot or cold, and asking to go to the bathroom. All other items were considered observational, including the other indicators of temperature. Hunger and thirst were considered as both verbal and observational, as both methods have been used in their determination.

### 2.4. Statistical Analyses

Descriptive statistics were used to document responses on the SODS per category or source of discomfort and per type of item. Correlational statistics were used to show associations between discomfort scores and cognitive, functional, and pain status.

## 3. Results

Participants had a mean of around 1 source of discomfort from each of the three categories (physical, body position, and environmental; see Table 2), with each participant experiencing between 0 and 10 indications. Three quarters of the sample experienced at least one physical source of discomfort, two-thirds experienced a discomfort associated with body position, and close to half experienced an environmental source of discomfort (Table 2). Most of the SODS items and related experiences are based on observable items, with relatively few being based on verbal responses of participants.

*The Relationship between SODS Categories and Cognitive, Functional, and Pain Status*. Pearson's correlations between indicators of cognitive function (long term memory and ability to understand others from the MDS as well as MMSE score), ADL (from the MDS), and PAINE with categories of SODS as well as types of information of SODS (verbal versus observational) are displayed in Table 3. Significant correlations were found between the cognitive indicators and the verbal items (*r* = −.30, *p* < .01 with long term memory, *r* = −.19, *p* < .05 with ability to understand others, and *r* = .21, *p* < .01 with MMSE score; see Table 3), supporting the hypothesis that those with higher levels of cognitive function would be more likely to respond and therefore to have an indicator based on those responses. However, neither the observable items nor the total SODS were correlated with any of the indicators of cognitive function. Poorer functional status as rated by the MDS based ADL score was most highly correlated with assessed discomfort related to body position (*r* = .40, *p* < .01) and was also significantly correlated with total SODS score (*r* = .28, *p* < .01), and with environmental sources of discomfort (*r* = .17, *p* < .05). These findings are mostly based on observable items of SODS which correlated significantly with poor ADL function (*r* = .30, *p* < .01). Pain as measured by the PAINE was correlated with sources of discomfort related to body position (*r* = .42, *p* < .01), but not significantly with other categories of SODS. Association was also reflected by correlations of pain with total SODS and with the observable items on SODS (*r* = .29 and .28, resp., *p* < .01 for both).

## 4. Discussion

This paper demonstrates a relatively straightforward and practical method to detect discomfort, its manifestations, and reasons for it in a population with advanced dementia. Our observations revealed a very high prevalence of discomfort in this population. Up to ten sources of discomfort, with a mean of 3 sources per person, were noted during 3-4 short observation periods. These sources relate to physical sources of discomfort, uncomfortable body position, and inadequate environment. The methodology, in the case of many of the sources of discomfort, also provides a directive for the caregiver as to how to offer an intervention.

In regard to our hypotheses, results are consistent with the majority. Specifically, the results are in agreement with the hypothesis that verbal items would be correlated with measures of cognitive function (MMSE and items from the MDS), but the observational items would not. Total SODS score was not related to cognitive functioning, a finding in which SODS differs from many other pain and discomfort scales.

The second hypothesis that body position indicators would correlate with poorer ADL was also in line with our findings. However, the third hypothesis that physical discomfort will be more closely related to pain than other types of discomfort was not supported by the findings. This may be due to the transitory nature of many of the sources of discomfort in this category (e.g., thirst, hunger, wanting to go to the bathroom) and to the nonpain nature of the most common item in this category, that is, being sleepy or tired and not in bed. In contrast, the body position category was most highly correlated with pain. While it is likely that many of the body positons described are painful, future study will need to untangle this relationship. It is possible that sitting in the same place for hours without moving may be painful, as may be the case with many of the other body position states described in the SODS. Yet, the relationship between pain and body position could take various forms of cause and effect; this needs to be further explored.

Although there is a significant relationship between pain and discomfort, they are clearly distinct constructs, with a Pearson correlation lower than .3. Only the body position category was significantly correlated with pain. By the nature of the SODS assessment, not only does it detect discomfort, but also it provides suggestions for alleviating the discomfort. Furthermore, it can also cast light on methods of care and systemic issues that need to be addressed to reduce the incidents of discomfort, thus illuminating the needs of PWD which were not previously assessed in a systematic manner.

The examination of the relationships between SODS and cognitive and functional status serves to validate the scale, to point to its limitations, and to suggest domains for future study. The relationship between functional limitations and body position and environmental sources of discomfort highlights the fact that with functional decline PWD become less able to position or reposition themselves comfortably or to obtain the means to remedy discomfort, such as getting a drink. There is also a decrease in this population in the ability to verbalize their need or to request help, as demonstrated by the relatively low prevalence of positive responses to verbal items. Being cognizant of the bias introduced by reliance on verbal responses of persons with advanced dementia, the SODS is largely observational and does not require verbal output from participants. Indeed, the total score did not correlate with cognitive functioning.

Results are limited by the fact that participants were recruited only if they were reported to manifest agitated behaviors. Since such behaviors are often manifestations of unmet needs, it is possible that this population has higher levels of sources of discomfort than other persons with dementia. On the other hand, the reasons for these sources of discomfort, that is, the inability to care for oneself or to express the discomfort verbally, are true for all populations with advanced dementia. Future studies validating the SODS, possibly with some additional items, in different samples are recommended.

In developing the SODS, we focused on observable and nonpain signs of discomfort. Yet, it is likely that the assessment could be improved by examining additional symptoms which may indicate discomfort. Some clear candidates for inclusion in future versions of the SODS are high levels of noise in the environment, as well as nonverbal signs that the person needs to go to the bathroom or needs a change of underwear. Future research should also examine which behavioral indicators, if any, are associated with discomfort. In doing so, it may examine the utility of adding items such as burping, wheezing, coughing, sweating, or labored breathing to the SODS. While most of the items likely represent discomfort across all participants, for example, sitting in the same position and place for over two hours, others may benefit from individualization. For example, some persons may like feeling hot or being in a hot environment. To what extent is it possible to determine such preferences in this population and to what extent does knowing those preferences have sufficient impact on ratings to warrant the additional effort of obtaining them are topics for future investigation.

## 5. Conclusions

Identifying sources and indications of discomfort, not only pain, is crucial to understanding discomfort in PWD. Based on this understanding, care modifications can be developed to address these discomforts. Using the SODS allows for exploration of discomfort beyond what has been available through previous assessments and to broaden the understanding of what causes discomfort. For future study, it may be interesting to correlate the quality of nursing home care with the number and types of sources of discomfort in PWD, as quality and quantity of care do likely impact comfort levels.

## Figures and Tables

**Table 1:** Participants' characteristics.

	*N* = 179
	M (SD)/%
Background	
Age	86.08 (8.62)
Gender (female)	72.1
Ethnicity	
Caucasian	74.3
African American	13.4
Asian/Pacific Islander	7.8
Hispanic/Latino	3.4
Other	1.1
Marital status	
Widowed	58.4
Married	24.3
Separated/divorced	9.2
Never married	7.5
Education	
High school or lower	60.7
College/technical school	23.4
Graduate degree	16.0
Function	
Cognitive status (MMSE)	8.79 (6.44)
ADL (MDS, mean of 10 items, scale 0–4)	2.72 (.92)
Vision (MDS) impaired	33.7
Hearing (MDS) difficulties or impairment	27.9
Diagnoses and medication	
Diagnosis index (of 11 disease categories)	5.32 (1.38)
Total number of medications	8.30 (2.40)
% administered	
Sedatives	11.9
Antipsychotics	53.6
Antidepressants	62.0
Antianxiety medications	33.9
Analgesics	98.3

ADL: Activities of Daily Living from 0 (independent) to 4 (total dependence); MMSE: Mini Mental State Examination, range 0–30; higher scores indicate higher cognitive function.

**Table 2:** Distribution of categories of sources and indicators of discomfort based on the Sources of Discomfort Scale.

Category	Number of indicators/items^a^ in category	% of participants manifesting the category	Average number of sources/indicators/items (SD)	Range of sources/indicators/items
Physical sources of discomfort	8	75%	1.04 (.89)	0–5
Body Position	6	66%	1.02 (.97)	0–4
Environmental sources	6	46%	.92 (.96)	0–4
Total SODS	20	91%	2.98 (2.11)	0–10
Type of item				
Verbal items	6	21%	.29 (.62)	0–3
Observable items	20	90%	2.79 (2.00)	0–8

^a^Note: items refer to specific questions, whereas indicators refer to sources of discomfort. For example, 3 items on the SODS address the indicator of cold temperature. The first 4 lines of the table refer to indicators, whereas the last two refer to items.

**Table 3:** Correlations of functional and pain variables with categories of sources of comfort and with type of SODS item.

	Long term memory (from MDS)^a^	Ability to understand others (from MDS)^a^	MMSE score^b^	ADL^a^	PAINE^c^
Category					
Physical sources of discomfort	−.137	−.138	.043	.055	.204
Body position	−.083	−.041	−.123	.399^*∗∗*^	.424^*∗∗*^
Environmental sources	.034	−.108	−.018	.170^*∗*^	−.021
Total SODS	−.082	−.126	−.046	.283^*∗∗*^	.287^*∗∗*^
Type of item					
Verbal items	**−**.302^*∗∗*^	**−**.187^*∗*^	.212^*∗∗*^	.053	.071
Observable items	−.009	−.097	−.096	.296^*∗∗*^	.276^*∗∗*^

^*∗∗*^Correlation is significant at the 0.01 level (2-tailed).

^*∗*^Correlation is significant at the 0.05 level (2-tailed).

^a^Higher scores indicate poorer function.

^b^Higher scores indicate better function.

^c^Higher scores indicate more pain.
